# Retrospective analyses of trends in pancreatic surgery: indications, operative techniques, and postoperative outcome of 1,120 pancreatic resections

**DOI:** 10.1186/s12957-015-0525-6

**Published:** 2015-03-12

**Authors:** Uwe A Wittel, Frank Makowiec, Olivia Sick, Gabriel J Seifert, Tobias Keck, Ulrich Adam, Ulrich T Hopt

**Affiliations:** Clinic of General and Visceral Surgery, Department of Surgery, Universitätsklinik Freiburg, Hugstetter Str. 55, 79106 Freiburg, Germany

**Keywords:** Pancreatic surgery, Pancreatic resections, Indication, Morbidity, Mortality

## Abstract

**Background:**

Hospital volume, surgeons’ experience, and adequate management of complications are factors that contribute to a better outcome after pancreatic resections. The aim of our study was to analyze trends in indications, surgical techniques, and postoperative outcome in more than 1,100 pancreatic resections.

**Methods:**

One thousand one hundred twenty pancreatic resections were performed since 1994. The vast majority of operations were performed by three surgeons. Perioperative data were documented in a pancreatic database. For the purpose of our analysis, the study period was sub-classified into three periods (A 1994 to 2001/*n* = 363; B 2001 to 2006/*n* = 305; C since 2007 to 2012/*n* = 452).

**Results:**

The median patient age increased from 51 (A) to 65 years (C; *P* < 0.001). Indications for surgery were pancreatic/periampullary cancer (49%), chronic pancreatitis (CP; 33%), and various other lesions (18%). About two thirds of the operations were pylorus-preserving pancreaticoduodenectomies. The frequency of mesenterico-portal vein resections increased from 8% (A) to 20% (C; *P* < 0.01). The overall mortality was 2.4% and comparable in all three periods (2.8%, 2.0%, 2.4%; *P* = 0.8). Overall complication rates increased from 42% (A) to 56% (C; *P* < 0.01).

**Conclusions:**

Mortality remained low despite a more aggressive surgical approach to pancreatic disease. An increased overall morbidity may be explained by more clinically relevant pancreatic fistulas and better documentation.

## Background

One major advance in pancreatic surgery over the past two decades is the resection of premalignant pancreatic lesions to prevent development of pancreatic cancer [1]. This is only possible because pancreatic resections have developed into routine procedures with low mortality [2]. Many factors like hospital volume, surgeons’ experience, and improved management of postoperative complications are known to improve the outcome of pancreatic surgery [3]. At the same time, pancreatic resections are performed in patients with a higher perioperative risk profile.

In our study, we analyze patient characteristics and shifts in surgical strategies in pancreatic resections in our clinic. Additionally, we examine the morbidity and mortality during 18 years of surgical practice in a collective of 1,120 patients who underwent resections of the pancreas in a single institution cohort.

## Methods

From July 1994 to September 2012, 1,120 patients underwent pancreatic resections for various reasons at our institution. Pancreatic resection included pylorus-preserving pancreatic head resections, classical Whipple procedures, distal pancreatectomies, total pancreatectomies, segmental resections, duodenum-preserving pancreas head resections, and central pancreatic resections. For the purpose of analysis, three time periods were defined. Period A includes patients treated from July 1994 to August 2001, period B includes patients from September 2001 to December 2006, while period C includes patients from January 2007 to September 2012. The entire team working with the senior author UTH moved from the University of Rostock to the University of Freiburg in September 2001 with continuity in staff and experience.

Operative specimens underwent standard histopathological evaluation. This included documentation of tumor size, lymph node status, and resection margin. A negative resection margin was defined as tumor distant to the resection margin independent of the precise distance.

### Data collection and statistics

Perioperative data was collected in a SPSS database (SPSS, Version 21.0, SPSS Inc., Chicago, IL, USA) in accordance with the Helsinki guidelines and approved by the local committee. Data analyses were performed in a retrospective manner. Descriptive statistics were generated. The median age and BMI as well as the corresponding 95% confidence intervals were calculated. To investigate the amount of blood transfusions, the average volume and the corresponding 95% CI were calculated. Significant alterations between the groups were determined by chi-square test according to Pearson.

## Results

Perioperative data was collected from 1,120 consecutively treated patients of our institution from 1994 until 2012.

### Patient characteristics

Throughout the observed 18 years, a significant increase in age was observed (Table 1). While the median age was 51 (15 to 77) years during period A, the age increased gradually to 64.9 (17 to 89) years in period C. Distribution of gender shows a shift from male to female which is due to the higher prevalence of patients with chronic pancreatitis during period A. This also explains the observed shift from benign to malignant diagnoses. Epidemiologically, this may be explained by epidemiological differences in disease prevalence of chronic pancreatitis between North and South Germany.

**Table 1:** **Patient characteristics**

	**1994 to 2001**	**2001 to 2006**	**2007 to 2012**	***P*** **value**
Number of resections	363	305	452	
Gender	Male 245 (67.5%)	Male 158 (51.8%)	Male 248 (54.9%)	<0.001
	Female 118 (32.5%)	Female 147 (48.2%)	Female 204 (45.1%)	
Age	51 (15 to 77)	64 (9 to 84)	65 (17 to 89)	<0.001
Body mass index	22.8 (14 to 35)	23.6 (15 to 36)	24.4 (15 to 41)	<0.001
Nicotine	Not determined	Yes 56 (31.6%)	Yes 121 (27.5%)	Not significant
		No 121 (68.4%)	No 319 (72.5%)	
		Not determined 128	Not determined 12	
ASA physical status	Not determined	I 13 (7.7%)	I 30 (6.9%)	Not significant
		II 106 (62.7%)	II 242 (55.8%)	
		III 49 (29.0%)	III 153 (35.3%)	
		IV 1 (0.6%)	IV 9 (2.1%)	
		Not determined 136	Not determined 18	
Dignity of disease	Benign 226 (62.3%)	Benign 109 (35.7%)	Benign 163 (36.1%)	<0.001
	Malignant 137 (37.7%)	Malignant 196 (64.3%)	Malignant 289 (63.9%)	

Number (percentage) or median (95% CI). ASA, American Society of Anesthesiologists.

### Pathologic diagnosis

Geographical shifts in disease prevalence explain the drop of patients with chronic pancreatitis from 56.2% during period A to 17.3% during period C (Table 2). In part, this also explains the higher relative prevalence of patients with malignancies, which increased from 22.3% to 42.3% during the observation period. Ductal adenocarcinoma was by far the most frequently underlying pathology in patients with malignancies, followed by ampullary carcinomas. A shift of disease prevalence within the collective of patients with malignant diagnoses was not observed. Increasing understanding of benign and premalignant cystic pancreatic lesions led to increased resection rates of intraductal papillary mucinous neoplasms (IPMNs), beginning at 0% between 1994 and 2001 and increasing to 7.7% during the third period until 2012. Additionally, improved preoperative diagnostic possibilities and further data on premalignant cystic lesions lead to an increased rate of resections of rare pancreatic lesions.

**Table 2:** **Histologic diagnosis**

	**1994 to 2001**	**2002 to 2006**	**2007 to 2012**
Malignoma			
Ductal adenocarcinoma	81 (22.3%)	108 (35.4%)	191 (42.3%)
Distal bile duct cancer	13 (3.6%)	29 (9.5%)	15 (3.3%)
Ampullary carcinoma	25 (6.9%)	29 (9.5%)	34 (7.5%)
Duodenal carcinoma	2 (0.6%)	9 (3.0%)	4 (0.9%)
Pancreatic neuroendocrine tumor	6 (1.7%)	13 (4.3%)	29 (4.3%)
Metastases	4 (1.1%)	5 (1.6%)	9 (2.0%)
Benign tumors			
IPMN	0 (0%)	7 (2.3%)	35 (7.7%)
Chronic pancreatitis	204 (56.2%)	83 (27.2%)	78 (17.3%)
Others	28 (7.7%)	22 (7.2%)	57 (12.6%)

Number (percentage). IPMN, intraductal papillary mucinous neoplasm.

### Surgical procedures and surgeon experience

Eighty-one percent of procedures were performed by three main surgeons. The caseload of these surgeons was 291, 292, and 317, respectively. Two hundred twenty pancreatic resections were performed by less experienced surgeons. The annual number of resections of the pancreas increased gradually from an average of 51 resections per year during the years 1994 to 2001 to 57 per year from 2001 to 2006 up to 95 resections per year from 2007 to 2012 (Table 3).

**Table 3:** **Surgical procedures and experience of surgeon**

	**1994 to 2001**	**2002 to 2006**	**2007 to 2012**	***P*** **value**
Resections				
Annual number of resections	51	57	95	Not significant
Whipple procedure	51 (14.0%)	23 (7.5%)	35 (7.7%)	Not significant
Pylorus-preserving pancreaticoduodenectomy	193 (53.2%)	180 (59.0%)	259 (57.3%)	Not significant
Duodenum-preserving pancreatic head resection	67 (18.5%)	41 (13.4%)	16 (3.5%)	Not significant
Distal pancreatectomy	42 (11.6%)	52 (17.0%)	105 (23.2%)	Not significant
Total pancreatectomy	5 (1.4%)	7 (2.3%)	30 (6.6%)	<0.001
Segmental resection	4 (1.1%)	2 (0.7%)	4 (0.8%)	Not significant
Central resection	0 (0%)	0 (0%)	3 (0.6%)	Not significant
PV resection	28 (7.7%)	59 (19.3%)	89 (19.7%)	<0.001
Laparoscopic procedure	0 (0%)	0 (0%)	53 (11.7%)	<0.001

Number (percentage). PV, portal vein.

Due to the reduced rate of patients with chronic pancreatitis, the frequency of duodenum-preserving resections decreased from 18.5% to 3.5%. The rate of pancreaticoduodenectomies remained unaltered, while the rates of total pancreatectomies and distal pancreatectomies increased from 1.4% to 6.6% and from 11.6% to 23.2%, respectively. The rate of venous resections increased from 7.7% in period A to 19.7% in period C.

Laparoscopic resections of the pancreas were introduced during period C. In period C, 10% of resections were performed in a laparoscopically assisted technique. In these cases of pancreaticoduodenectomy, the resection was performed in a laparoscopic manner while reconstruction was achieved *via* mini laparotomy. Distal pancreatectomies were performed as fully laparoscopic procedures.

### Morbidity and mortality

The mortality rates during all three periods were low at 2.8%, 2.0%, and 2.4%, respectively (Table 4). However, the surgery-related morbidity increased from 28.7% to 50.7%. Interestingly, the reoperation rate did not change significantly. An increased rate of pancreatic fistulas is one reason explaining the increase of morbidity. Partly, this is due to the relative increase of resections of pancreatic malignancies or premalignant lesions in patients with soft pancreatic texture, while there was a decrease of resections of patients with chronic pancreatitis. Additionally, the documentation of postoperative complications like pancreatic fistulas was improved due to the initiation of prospective studies.

**Table 4:** **Morbidity and mortality**

	**1994 to 2001**	**2002 to 2006**	**2007 to 2012**	***P*** **value**
Mortality	10 (2.8%)	6 (2.0%)	11 (2.4%)	Not significant
Overall morbidity	151 (41.6%)	170 (55.7%)	257 (56.9%)	<0.001
Operation-related morbidity	104 (28.7%)	111 (36.4%)	229 (50.7%)	<0.001
Reoperation	33 (9.1%)	33 (10.8%)	61 (13.5%)	Not significant
Transfusion				
Number of patients with transfusion	313 (87.5%)	66 (21.6%)	87 (19.3%)	<0.001
Average volume	1,523 (1,345 to 1,701) ml	170 (124 to 217) ml	177 (129 to 227) ml	<0.001
Pancreatic fistula grades A to C	37 (10.2%)	79 (25.9%)	180 (39.8%)	<0.001

Number (percentage) or average (95% CI).

### Morbidity and mortality in dependency of diagnosis

Morbidity and mortality were influenced by the histopathological diagnosis, and two distinct deviations were apparent (Table 5). The rate of reoperations was higher in patients with IPMNs when compared to patients with other pathohistological diagnoses. While the main reasons for reoperations in these patients were postoperative hemorrhage and leakage of the biliary anastomosis, a possibly increased risk for the leakage of the biliary anastomosis could be explained by the usually non-dilated bile duct in these patients. The second observation was that the rate of pancreatic fistulas was highest in patients with pancreatic neuroendocrine tumors and other pathohistological entities. This may be explained by the high rate of distal and atypical pancreatic resections in these patients.

**Table 5:** **Morbidity and mortality in dependency of diagnosis**

	**PDAC**	**Periampullary carcinoma**	**Chronic pancreatitis**	**pNET**	**IPMN**	**Other**	***P*** **value**
	**(** ***n*** **= 380)**	**(** ***n*** **= 160)**	**(** ***n*** **= 360)**	**(** ***n*** **= 48)**	**(** ***n*** **= 42)**	**(** ***n*** **= 125)**	
Mortality	16 (4.2%)	4 (2.5%)	5 (1.4%)	0 (0.0%)	2 (4.8%)	0 (0.0%)	<0.05
Overall morbidity	194 (51.1%)	101 (63.1%)	161 (44.1%)	22 (45.8%)	25 (59.5%)	75 (60.0%)	<0.001
Operation-related morbidity	131 (34.5%)	76 (47.5%)	121 (33.2%)	23 (47.9%)	23 (54.8%)	70 (56.0%)	<0.001
Reoperation	40 (10.5%)	21 (13.1%)	35 (9.6%)	6 (12.5%)	11 (26.2%)	14 (11.2%)	<0.05
Pancreatic fistula grades A to C	70 (18.4%)	62 (38.8%)	68 (18.6%)	23 (47.9%)	15 (35.7%)	58 (46.4%)	<0.001

Number (percentage). PDAC, pancreatic ductal adenocarcinoma; pNET, primitive neuroectodermal tumor; IPMN, intraductal papillary mucinous neoplasm.

## Discussion

Our data mirrors several epidemiological developments and the expansion of indications of pancreatic resections over the past 18 years. One very interesting insight provided by this study is the correlation between the dramatic change of the prevalence rates of chronic pancreatitis with the geographical relocation of the main surgeons. Such regional shifts have been observed in the past and are believed to be linked to nutritional habits as well as differences in alcohol and possibly nicotine consumption [4,5]. Therefore, the rate of pancreatic resections due to benign diseases was higher during period A when compared to period C. For the same reason, the age of the patients increased over time since patients suffering from chronic pancreatitis have a median age in the 40s, and the age of patients with pancreatic cancer averages in the 60s [6,7].

The analysis of the diagnosis obtained after pancreatic resections sheds light on several developments. For one, it confirms the increase in pancreatic cancer cases mentioned above. Furthermore, the number of pancreatic resections performed due to premalignant lesions increased substantially from 0% to 7.7%. This occurred due to accumulation of data on benign cystic pancreatic lesions and subsequently adjusted treatment guidelines developed during the observation period, such as the Sendai guidelines [8]. However, even before the publication of these guidelines in 2006, an increase in pancreatic resections due to IPMNs was observed between 2001 and 2006. This indicates that even before the publication of these consensus guidelines, resections of premalignant lesions were regularly performed.

In terms of surgical procedures, duodenum-preserving techniques decreased substantially due to a reduced number of chronic pancreatitis cases, while at the same time, the number of distal pancreatectomies and total pancreatectomies increased substantially. Similar to other patient collectives, the increase in pancreatectomies was mostly due to an increased number of resections of main-duct-type IPMNs and the resection of larger carcinomas located in the pancreatic body [9]. The increase of distal pancreatectomies was mostly due to increased resections of not only benign but also malignant pancreatic lesions.

During the first two periods from 1994 until 2006, no laparoscopic resections were performed in our center. During the last period, there was a substantial increase in laparoscopic procedures. These included distal pancreatectomies with and without preservation of the spleen as well as laparoscopy-assisted pancreaticoduodenectomies. In the latter cases, the reconstruction was performed *via* mini laparotomy in contrast to other groups [10,11].

While mortality rates and the rate of reoperations remained low, the morbidity increased over time. Several factors are responsible for the increased rate of complications, both surgical complications as well as complications not directly linked to surgery. Most surgery-related complications were pancreatic fistulas and delayed gastric emptying. The greater number of pancreatic fistulas is due to several developments: the resection of more patients with non-malignant disease, fewer chronic pancreatitis cases, better documentation of fistulas, and publication of international guidelines in 2005 [12]. Secondly, an increase in delayed gastric emptying was observed which occurred due to the implementation of pancreaticogastrostomies for pancreatic reconstruction which are associated with increased rates of delayed gastric emptying compared to pancreaticojejunostomies [13]. A further increase in morbidity can be explained by the increased patient age and a higher rate of non-surgery-related complications. Furthermore, there was an increase of resections of advanced malignancies which can be observed by an increase in portal vein resections. The latter was not associated with a higher morbidity in our collective, while larger studies show that venous resection is associated with increased morbidity rates [14].

## Conclusions

We are able to demonstrate that pancreatic resection at a high-volume center is a safe procedure with a low mortality rate and low rates of reoperations. Operative mortality in our high-volume institutional series of more than 1,100 pancreatic resections remained low despite a more aggressive surgical approach to pancreatic disease. However, pancreatic resections still have a substantial morbidity that is mainly driven by the occurrence of pancreatic fistulas. The rate of surgical complications, however, is responsible for increased length of hospital stay and increased rates of readmission and therefore is the main driving force of increased treatment costs of patients undergoing pancreatic resections [15].
